# Special Issue: Cancer Metastasis and Therapeutic Resistance

**DOI:** 10.3390/biomedicines11051347

**Published:** 2023-05-03

**Authors:** Elizabeth S. Yeh

**Affiliations:** 1 Department of Pharmacology and Toxicology, Indiana University School of Medicine, Indianapolis, IN 46202, USA; esyeh@iu.edu; 2 Melvin and Bren Simon Comprehensive Cancer Center, Indiana University School of Medicine, Indianapolis, IN 46202, USA

**Keywords:** cancer, metastasis, therapeutic resistance

## Abstract

Metastasis and resistance to cancer therapeutics are critical barriers to curing cancer. This special issue entitled “Cancer Metastasis and Therapeutic Resistance” contains nine original contributions. The articles span a variety of human cancers, including breast, lung, brain, prostate, and skin and touch upon significant areas of interest such as cancer stem cell function, cancer immunology, and glycosylation.

## 1. Review Articles

This special issue contains one review article by Kilmister et al., which touches on the important topic of cancer stem cells (CSC) [1]. These specialized cells are a primary source of therapeutic resistance [2]. As a result, this review covers cancer stem cell (CSC) interactions with the tumor microenvironment (TME) and discusses the dynamic communication between CSCs and components within the tumor. These components, which include immune cells, contribute to the creation of a TME that promotes treatment resistance and metastasis. The authors discuss the relationship of CSCs to immune cells, the renin-angiotensin system, and convergent signaling pathways, including the Wnt/β-catenin, Notch, and Sonic Hedgehog (SHH) signaling pathways. Understanding these key mechanisms underlying treatment resistance and metastasis that are driven by CSC–TME interactions is important for the development of effective cancer treatments.

## 2. Research Articles, Brief Reports, and Communications

Sriratanasak et al. also tackle important aspects of CSC regulation in a research article that examines the impact of cisplatin-induced senescence on lung cancer cells [3]. Typically, senescence can be viewed as a terminal cell state, but prior findings suggest that senescence induced by therapeutic agents leads cancer cells to have a high tumor-initiating ability (reviewed in [4,5]). Findings from Sriratanasak et al. demonstrate that cisplatin-induced senescence caused Glucose-regulated protein 78 (GRP78) to bind to the ER-co-chaperone MTJ1, and an increase in the expression of stemness markers, including CD44, CD133, Nanog, Oct4, and Sox2 [3]. GRP78 signals through the PI3K-Akt signaling pathway are associated with CSC regulation. In future studies, it will be interesting to determine whether the GRP78-PI3K-Akt axis directly regulates the CSC function or if the findings are associative.

This issue had a significant set of original research, tackling different aspects of breast cancer. The article by Barutello et al. examines a combination of methods to target the cystine-glutamate antiporter xCT and p53 using the drug APR-246, which restores the wild-type function of p53 in mutant p53 tumors [6]. xCT is an emerging target in breast cancer that ties cancer cell metabolism to redox regulation [7]. Findings from Barutello et al. suggest that the combined targeting of xCT and p53 is effective at decreasing breast cancer cell viability and CSC self-renewal [6]. Furthermore, to study the in vivo effect of the combined targeting of xCT and p53, the authors immunized mice against xCT and co-treated them with APR-246. The results show that combined targeting was more effective than the individual targeting of xCT or p53.

The research article by Cheng et al. looks more distinctly at guanylate binding protein 5 (GBP5) and its expression in triple-negative breast cancer (TNBC) [8]. This study demonstrates that GBP5 knockdown impacts cell migration, PD-L1 expression, and the activation of interferon-gamma (IFN-g) and TNF-a signaling cascades. Furthermore, this study examined the potential for GBP5 to serve as a marker for poor prognosis and additionally looked at the combined effect of higher GBP5 and PD-L1 levels. The findings showed that high GBP5 and PD-L1 were associated with reduced brain metastasis-free survival.

Prior studies have shown that fucosylation, a type of glycosylation, promotes metastatic phenotypes and signaling in breast cancer cells [9,10]. Doud et al. contributed a communication piece using global proteomics to assess metastatic breast cancer cells in the presence or absence of a fucosylation inhibitor [11]. In this study, pathway analysis indicated the association of fucosylation with NF-κB and TNF signaling pathways.

Rhosin is a RhoGTPase RhoA/C-yes associated protein (YAP) pathway inhibitor [12]. Tsubaki et al. investigated the efficacy of Rhosin using metastatic melanoma and breast cancer cells [13]. These findings suggest that rhosin inhibits lung metastasis in vivo using mouse models of metastasis. The study further examines the mechanisms by which rhosin exerts its effects. Rhosin reduced RhoGTPase activity and YAP activity. The treatment of cells with rhosin inhibited tumor cell adhesion by suppressing the expression of the hyaluronan-mediated motility receptor RHAMM and inhibiting stromal-derived factor SDF-1-induced cell migration and invasion in a CXCR4-related manner.

Penas-Martínez et al. provided an interesting brief study about the effect of antithrombin (AT) on glioblastoma [14]. Recently, this same research group showed that AT has an anti-tumor effect on GB [15]. Interestingly, AT has different conformations; native, heparin-activated, prelatent, latent, and cleaved, each with different affinity properties to heparin. The present study expands prior findings by showing how a specific conformational form of AT, prelatent, is effective in impairing cell migration and invasion. This finding is interesting since the prelatent form of AT has a binding affinity that is less than native AT.

Spine magnetic resonance imaging (MRI) can be typically used to guide treatment for vertebral metastasis. Pichon et al. aimed to improve imaging techniques for metastatic breast and medullary thyroid cancers with metastasis of the spine [16]. The study authors propose to develop and use a technique called pre-targeted immuno-PET (iPET), which uses an anti-carcinoembryonic antigen (CEA) to guide PET imaging. These findings suggest that iPET may offer complementary information to MRI and provide a more precise mapping of vertebral segments with metastatic disease.

Iuliani et al. contributed a brief report investigating the effect of an agent abiraterone (ABI), which is a selective inhibitor of androgen biosynthesis, on the osteoblast secretome [17]. These studies build upon prior studies that indicate how ABI demonstrated survival benefits in metastatic castration-resistant prostate cancer (mCRPC) patients. Prior studies by this group using in vitro models indicated the direct effect of ABI on osteoclast and osteoblast function. The present studies expanded these findings using proteomic analysis to investigate the effects of ABI on the osteoblast cell secretome. These findings show how ABI had an effect on modulating the expression of key osteoblastic and osteoclastic gene markers.

## 3. Closing Remarks

Articles in this special issue span many topics, all aimed to increase knowledge in the area of metastasis and therapeutic resistance. Common themes include areas of significance in understanding the CSC function as well as breast cancer biology. The overall aim of this issue is to provide a broad representation of research areas that examine mechanisms and approaches for confronting metastasis and the failure of cancer treatments.
